# The Application of Cameras in Precision Pig Farming: An Overview for Swine-Keeping Professionals

**DOI:** 10.3390/ani11082343

**Published:** 2021-08-09

**Authors:** Elanchezhian Arulmozhi, Anil Bhujel, Byeong-Eun Moon, Hyeon-Tae Kim

**Affiliations:** Department of Bio-Systems Engineering, Institute of Smart Farm, Gyeongsang National University, Jinju 52828, Korea; mohanachezhian@yahoo.com (E.A.); anil.bhujel@gmail.com (A.B.); be25moon@naver.com (B.-E.M.)

**Keywords:** early disease detection, pig behavior, precision livestock farming, pig identification, cameras

## Abstract

**Simple Summary:**

The preeminent purpose of precision livestock farming (PLF) is to provide affordable and straightforward solutions to severe problems with certainty. Some data collection techniques in PLF such as RFID are accurate but not affordable for small- and medium-sized farms. On the other hand, camera sensors are cheap, commonly available, and easily used to collect information compared to other sensor systems in precision pig farming. Cameras have ample chance to monitor pigs with high precision at an affordable cost. However, the lack of targeted information about the application of cameras in the pig industry is a shortcoming for swine farmers and researchers. This review describes the state of the art in 3D imaging systems (i.e., depth sensors and time-of-flight cameras), along with 2D cameras, for effectively identifying pig behaviors, and presents automated approaches for monitoring and investigating pigs’ feeding, drinking, lying, locomotion, aggressive, and reproductive behaviors. In addition, the review summarizes the related literature and points out limitations to open up new dimensions for future researchers to explore.

**Abstract:**

Pork is the meat with the second-largest overall consumption, and chicken, pork, and beef together account for 92% of global meat production. Therefore, it is necessary to adopt more progressive methodologies such as precision livestock farming (PLF) rather than conventional methods to improve production. In recent years, image-based studies have become an efficient solution in various fields such as navigation for unmanned vehicles, human–machine-based systems, agricultural surveying, livestock, etc. So far, several studies have been conducted to identify, track, and classify the behaviors of pigs and achieve early detection of disease, using 2D/3D cameras. This review describes the state of the art in 3D imaging systems (i.e., depth sensors and time-of-flight cameras), along with 2D cameras, for effectively identifying pig behaviors and presents automated approaches for the monitoring and investigation of pigs’ feeding, drinking, lying, locomotion, aggressive, and reproductive behaviors.

## 1. Introduction

### 1.1. The Key Drivers of Adaptations in Precision Farming

As projected by economic experts, the world population in 2050 will be almost 10 billion, 23% more than the current population. Moreover, there will be a considerable shortfall in feeding the additional 3 billion people due to the lower food production achieved by conventional farming, as per statistical reports [1,2,3]. With 350 million tons of meat consumed per year worldwide, meat production needs to be boosted significantly, with less land use, to avoid hunger in the coming decades. Pork meat is the meat with the second-largest overall consumption, and chicken, pork, and beef together account for 92% of global meat production. Therefore, it is necessary to adopt more progressive methodologies such as precision livestock farming (PLF) rather than traditional farming methods such as uncontrolled environmental/microclimate, non-precision practices to improve production [4,5,6,7]. In the 2020s, some countries practice traditional farming methods wherein expensive human resources monitor the animals. The health and welfare of animals continue to be the primary goal of farms. Watching the boundaries and training the animals can be affordable using humans, but in terms of welfare, human monitoring is limited to identification with the naked eye; therefore, it is expensive and can miss key indicators [2,4,6,7]. For instance, the body temperature of an animal is a reproduction activity measurement, and temperature regulation is an important way to maintain the homeostasis of the animals. Measuring rectal temperature is a well-known technique for calculating animals’ body temperature; furthermore, the respiration rate and heart rate are also monitored according to human resource availability. Such methods are very labor intensive, but technological developments could reduce the human workload involved. When attempting to measure temperature using technology rather than traditional methods, temperature-sensing ear tags (which can also be used to detect heat stress), wearable and implantable devices, thermal imaging cameras, IR sensors, etc., can be used but thus far have been deployed in a limited fashion and so are not discussed in full in the literature [4,8,9]. 

In the mid-1980s, sophisticated agricultural strategies such as PLF were a major topic of discussion in scientific literature. PLF was considered impossible in underdeveloped countries. In this century, when technology use is at an all-time high, more than 50% of farmers in developing countries use the Internet and not just smartphones. In addition, PLF adaptation has advanced in recent years due to the emergence of advanced technologies such as affordable sensors, actuators, microprocessors, IoT-based monitoring systems, and big data analytics. The global precision livestock market’s worth was $3.1 billion in 2020, and it is expected to grow to $4.8 billion by 2025 at a combined annual growth rate (CAGR) of 9.0% [10]. In recent years, PLF for pigs has been in high demand due to an increase in mortality rate at every stage of the lifespan due to environmental effects on piglets and health issues such as heat stress, breathing problems, etc., that occur more often in the growing stage. Consequently, welfare assessment within all phases of swine production is mandatory to produce desirable outcomes. Moreover, consumers also demand a higher-welfare environment for sows and piglets in food production as standard practice. Many studies have reviewed the literal stakes and promises of the PLF system, and the following notes summarize the advantages of PLF adaptation over traditional farming practices,

- Efficiency—sustainably utilizing resources by reducing waste (feed and water intake and waste could be monitored using cameras [11,12,13], flow meters, feed scales, etc. [4,8,14]).
- Early disease detection—diagnosis of activity changes using cameras with the help of software, disease detection using welfare parameters (heat stress assessed by cameras [15], accelerometers, IR sensors [4,14], etc.), and improvement of animal welfare (tail biting, fighting, etc.).
- Environmental management—protect the animals from environmental stressors (PLF could regulate environmental stressors such as temperature, humidity, airflow, ventilation, etc.) [3,14].
- Performance—the workload of the animals can be reduced, or they can be subjected to external stress due to human activity inside the pen [8,16].
- Information and individual monitoring—maximum data could be stored in a computer, which could be used to further visualize the situation (feeding efficiency can be calculated by collecting data of feed intake and the weight of the pigs [17,18,19]; body surface temperature data can be used to track heat stress [15]). All the data, including on physical movement, performance, biological information, phonotype properties, etc., can be used to visualize not only group- or house-level data but individual data, too [20,21,22].
- Analyzing ability—with big data, it is possible to produce advanced-level software to notify farmers about standard criteria [20,21,22].

### 1.2. Review Objectives

The purpose of PLF is to provide affordable and straightforward solutions to severe problems with certainty; some sensor systems such as RFID are accurate but not affordable for small- and medium-sized farms. Furthermore, many farmers have rejected PLF due to its complexity, their inexperience in handling technical devices, etc. On the other hand, camera sensors are commonly available and can easily collect information compared to other sensor systems in pig barns [4,12,15]. Moreover, cattle have a broader chance to conduct research rather than pigs. For instance, a neck or leg belt with sensors can be attached to the cow to obtain the biological data, activity, and movement information. Whereas in the case of pigs, such kinds of attachable belts except RFID are practically difficult because other pigs can bite/break the sensors since the pigs are aggressive and curious about new things. Therefore, cameras have the ample chance to monitor pigs. However, the lack of targeted information about the application of camera sensors in the pig industry is a shortcoming for swine farmers and researchers. So far, several studies have been conducted to identify, track, and classify behaviors and attempt the early detection of diseases in pigs using 2D/3D cameras. This review describes the state of the art in 3D imaging systems (i.e., depth sensors and time-of-flight cameras), along with 2D cameras, for effectively identifying pig behaviors, and presents automated approaches for the monitoring and investigation of pigs’ feeding, drinking, lying, locomotion, aggressive, and reproductive behaviors. In addition, the review summarizes the related literature and points out limitations to open up new dimensions for future researchers to explore.

## 2. Introduction to Cameras Used for PLF

Cameras are ubiquitous tools for science and have been accessible worldwide at an affordable price for at least the last two decades. There are many types of cameras, such as CCD cameras, infrared cameras, depth cameras, etc., available on the market; each type of camera offers different information and image parameters. However, the current review considers CCD, depth, and infrared cameras, which are commonly used in studies. Image acquisition is the first step of any image-based analysis and can be defined as capturing numeric information via cameras [6,15,23]. In the last two decades, images have been utilized to improve the welfare of animals by extracting information from those images. A CCD camera can capture two-dimensional (2D) color images of an object, which can be used for further analysis. Those images give information on color (RGB), texture [18,24], shape [25], width [18,26,27], height [6,28,29], pixel values [6,29,30,31], etc., in a numerical form. The RGB color band (red, green, and blue) can also produce gray, hue, saturation, and intensity factors by using image-processing techniques. Feature extraction is the next step for image processing: extracting the information, as mentioned earlier, according to particular needs. For instance, pixel values can correlate with real-time measurements of a pig to develop a simple program to find the length of pigs without measuring another pig’s length. Likewise, finding out the weight, girth, height, and other body information of pigs using 2D images can easily be achieved using image-processing techniques. Pigs are constantly growing in a group/herd; separating one pig’s information is also possible through image-processing techniques.

In recent years, many image-based studies have used depth sensors in various fields such as navigation for unmanned vehicles, human–machine-based systems, agricultural surveying, livestock monitoring, etc. [4,32]. Initially, depth sensors were used for distance measurement, but depth cameras were used for multiple purposes due to the development of advanced software. In simple terms, depth images are 2D images that have the distance values of the image pixel across a wide field-of-view (FoV) from the sensor coordinate system measured by the time-of-flight (TOF) concept. The depth is determined by emitting a pulse and then measuring the time differential for the light emitted to travel to an object and back to a detector. For instance, Lidar (light detection and ranging) distance sensors emit a laser or infrared signal with a field-of-view of up to 2°, which offers accurate information regarding a single distance [14,15]. Likewise, they can provide a 3D image using an infrared light source and a CCD detector. The depth information is limited to FoV, so that fluctuations in external factors such as light, background noise, etc., cause a relative error in depth sensors. Even though there is a high chance of producing data with noise, this can be tackled with calibration and preprocessing techniques. It is easier and cheaper to reconstruct a 3D approach with depth sensors compared to other 3D reconstruction techniques such as stereo vision or laser vision. Recent studies have used depth information to construct 3D images; those images are then used in facial recognition, activity identification, and other types of morphological identification [14,15,23]. 

Infrared cameras work on the same principle as typical RGB cameras; CCD cameras measure the radiation of visible bands; and thermal cameras detect characteristic near-infrared radiation (0.75–1.4 µm (micrometers)) and thermal radiation (8–15 µm) [9,15]. Initially, thermal imaging systems were used in the oil and gas industries, pharma industries, for defense purposes, etc.; later on, they were adapted to find the temperature of animals. Since pigs are homoeothermic animals, they emit heat through temperature variations that can be identified quickly using thermal images. Unlike CCD and depth cameras, thermal cameras are expensive; still, early detection is an important way to be aware of diseases and could be possible with thermal images. 

## 3. An Overview of Software Used to Analyze an Image or Video

The core of camera-based technologies is software; cameras can only capture images, whereas software can interpret the data for image-based studies. Therefore, basic knowledge of the software used for the camera-based studies provides a better understanding of the process. This section describes primary-level machine-learning concepts and the nomenclature for the algorithms. Algorithms are a process or set of rules to be followed in calculations and are the backbone of software. Machine vision/computer vision, known as a subset of artificial intelligence (AI), is mainly used for problems with image and video recognition, image analysis and classification, media recreation, recommendation systems, natural language processing, etc. [33,34,35,36]. Convolutional neural networks (CNN) are a deep-learning-based robust algorithm to fulfill machine vision tasks. Like the other artificial neural network (ANN) models, CNN also has layers, whereas general ANN models have an input layer, hidden layer, and output layers. CNN has a convolution layer where a multiple feature detector identifies different things such as edges, different shapes, bends, different colors, etc. Similar to the convolutional layer, the pooling layer also extracts the features that are rotational and positional to improve the efficiency of the model during the training time. The principal purpose of the polling layers is to decrease the computational cost required to process the data through dimensionality reduction. The fully connected layer is a flattened version of the pooling layer, and the features are changed to a one-dimensional (1D) array of numbers (or vector). One or more fully connected layers, also called dense layers, connect every input to every output by a learnable weight. The whole process is explained as features from an image/video extracted in the convolution layer, and then it is down sampled by pooling layers; they are then mapped by a subset of fully connected layers to create the final outputs of the network, such as the probabilities for each class in classification tasks [34,35,36].

Subsequently, there are various algorithms such as support vector machine (SVM), region-based CNN (R-CNN), mask R-CNN, faster R-CNN, you only look once (YOLO), etc., available based on CNN concepts. However, pre-trained CNN models are ready to use based on the particular problem. Unlike other CNN models, pre-trained models do not need to be developed from scratch since they were developed and trained using a considerable number of public and private datasets to be applicable to a particular problem [33,35,36]. For instance, GoogLeNet is an inception-based pre-trained model developed to classify more than 1000 objects and was trained with a large number of class object images. Therefore, if a person wanted to classify cats and dogs, they could use GoogLeNet directly since it has already been trained; therefore, the algorithm knows the features available for differentiating dogs and cats. However, a complex problem could be solved with a pre-trained model after the fine-tuning of optimizers, called transfer learning. MobileNet, Alexnet, GoogLeNet, VGG, and mobile net are some popular pre-trained models available on the market.

## 4. Identification of Pigs

### 4.1. Facial Recognition

The identification of livestock has been practiced for several centuries, and although it was a means of claiming ownership in the early days, it later played a significant role in the detection and control of veterinary infections in the herd [37]. Welfare [37,38], behavior identification [1,37], activity management of livestock [39,40], and public welfare [41] are reasons for needing to identify animals. A wide range of methods for the identification of livestock has been utilized so far. Techniques such as ear clipping, ear notching, ear tagging, microchipping, electronic identification devices (EID), and numbering or marking on pig skin have been practiced. An EID principled radio frequency identifier (RFID) is popular for identifying pigs. RFID is an advanced version of numbered ear tags, and passive electronic tags consist of a radio frequency identifier (RFID) that emits a signal to the reader through a microchip and a coiled copper antenna. Low frequency (LF: 125 kHz or 134.2 kHz), high frequency (HF: 13.56 MHz), and ultra-high frequency (UHF: 860–960 MHz) are the three primary frequency ranges in RFID systems [39]. Even though RFID has advantages such as a simple mechanism, low cost, and reliable correlations for identifying objects, the following qualities make it unsuitable for pig identification [39,42]:

(1) Range: RFID has a limited range (even long-range readers state a maximum distance of 120 cm) at which the tags can be activated and read successfully [39]. In addition, multiple tags cannot be read concurrently; therefore, the data may not be reliable since pigs are playful and bunch together.
(2) Readability: Ear-tagged RFID can become illegible for reasons such as wear and tear, breakage, and soiling [42].
(3) Loss: Tags may be lost due to ear tearing during fighting or playing [39]. This is possible since the pig barn has metal objects; in addition, pigs are playful with plastic objects. In addition, the RFID tags are often exposed to harsh environments with excessive dirt, dust, and moisture, and they must function in extreme heat and cold, from −30 °C to 70 °C [42].
(4) Welfare: Poor application of RFID could result in infection or ear damage. In addition, ear tags can be transferred from one animal to another, which increases infection possibilities [42].

In the early 21st century, cameras are able to identify human faces due to astounding advances in technology that may also provide an opportunity to correct discrepancies in animal identification [43]. Additionally, artificial intelligence software development has played a significant role in identifying livestock with cameras. The limitations of RFID in identifying animals have been overcome by facial recognition using cameras [40]. By adapting the algorithms that produce promising human facial recognition outcomes, successful pig facial identification has been demonstrated in previous studies [40,43]. Similar to the mechanism of human facial recognition, features such as the pig eye region [40,43], snout [40,43], wrinkles [44], and Euclidean distance [44] were extracted. The extracted features were used as input for the training and validation of algorithms. Hansen et al. [43] have demonstrated pig facial recognition by using fisher faces, a VGG-face model, and deep CNN algorithms with a final accuracy of 96.7%. Likewise, a deep CNN model was utilized to identify pig faces by Marsot et al. [40]. Marsot et al. have proven that such facial identification models are not limited by age; in addition, they can identify faces one month after the pictures or video are taken. Other researchers [40,43] used deep CNN models to identify 10–16 pigs at the farm level with more than 90% accuracy in less than 1 s (0.002 s per image) using 2D images. In the future, the accuracy of the algorithms could be improved by adding more training pictures, using RGB images, preprocessing before training (structural similarity index measure and image filtering), etc.

### 4.2. Live Weight Detection

Live weight is crucial for rearing pigs and chickens since the livelihood of farmers revolves around the weight of the animals [6,30,31,45]. Studies indicate that the cost of feeding a pig is 75% or more of the total production cost [6]. Periodic monitoring of pig weight is essential to optimize these costs since food intake and weight gain are linearly correlated and underfeeding or overfeeding issues could be revealed through this process [46,47]. Monitoring live weight is not only due to the uncertainty in feeding; it is an index for evaluating the quality of reproduction and the rate of growth [6,30], the reproductive period [48], feed conversion efficiency [47], and disease occurrence [46]. In addition, profit and loss accounts can be evaluated using the live weight concerning current market conditions [46].

Initially, the weight of pigs was assessed through observation by experts using eyes and hands, which was inaccurate compared to the actual values [5,6,7]. Later weight bands were used to find the live weight, but this was not only a cumbersome process; it also required a vast number of human resources along with an abundance of time [7,49]. Other commercial electronic weighing platforms or loading cells have been introduced to pig barns and used on many farms so far; still, loading cells are not affordable, and maintaining them is arduous due to the harsh environment imposed by the pigs. Such reasons mean they are not considered an optimal way for pig producers to find the live weight [6]. Alternatively, indirect methods such as calculating the body weight from the body dimensions (length and girth) were introduced in earlier studies [7,18,49], especially studies that mentioned bodyweight and heart girth. However, such a mechanism also required human resources and thus, may cause stress to humans and pigs, since pigs are sensitive to human handling.

Studies such as the one above proved a correlation between morphology and weight, despite the shortcomings. It provided the motivation to assess features by a contactless method. Later, in 1990, Schofield [6] implemented the indirect method by using a nondestructive method, a camera. That author captured images of a pig from side view and top view (using a mirror placed 45° perpendicular to the pen); he extracted information of length (tail to scapula), girth, height at back and shoulder, and width from 15 pigs (the same pig was captured at 30–80 kg). His study predicted the live pig weight by correlating all the features with a ±6.2% error rate. Similar to that study, many researchers [29,30,50] have utilized CCD cameras to assess features such as the length and width of pigs (length from scapula to snout, length from tail to scapula, shoulder width, breadth at middle, and breadth at back) and boundary area to calculate the live weight. For the periodic monitoring of animals in earlier studies [31,45], CCD cameras were operated in a top-view position, so that some features such as the height of the pig and the heart girth were challenging to acquire; therefore, new features such as the area, convex area, perimeter, eccentricity, major and minor axis length, and boundary detection were utilized by those studies. They extracted the features using preprocessing techniques such as edge detection, background removal, grayscale image conversion, and removing the tail and head, and such features were correlated with the bodyweight using ANN and VQTAM. 

Subsequently, 3D-based depth cameras were employed [24,51,52,53] to monitor the live weight and weight gain through a fully automatic system. Kinect sensors collect depth and infrared information preprocessed by morphological filtering and trained with DNN and faster R-CNN-based robust architectures. The relative error for the weight estimation, 0.374–4.6%, was obtained in those studies during the validation of 20–37 pigs from various weight ranges. Earlier studies raised hopes that those models could be implemented at the farm level with some fine-tuning. In addition, several products are available on the market to estimate individual weight using cameras through a mobile application or a computer. For instance, Weight-Detect (PLF-Agritech Europe, Edinburgh, UK), Pigwei (Ymaging, Barcelona, Spain), eYeScan (Fancom BV, Panningen, The Netherlands), Growth Sensor (GroStat, Newport, UK), OptiSCAN (Hölscher, Emsburen, Germany), and WUGGL One (WUGGL, Lebring, Österreich) are among the many weight solution products on the market [14]. 

### 4.3. Growth Patterns and Mass Calculation

As mentioned in the live weight section, body morphology is not only an indicator of bodyweight; additionally, it acts as an indicator of other critical measures such as consistency in growth [54], weakness in legs [55], carcass traits [56,57], and the health and welfare of animals [29,58]. Therefore, the adequate measurement of the growth of animals is necessary to produce quality meat to the end customer since the mass of the animal is essential for a quality carcass as per experts’ assessment. Therefore, the mass and volume of pigs are measured by the same morphological parameters used to detect the live weight. Furthermore, to assess the quality of the carcass, backbones and side ribs are also considered. Generally, a set of scoring categories, 1–5, is followed by carcass assessors based on the detection of ribs, backbone, H-bones, and pin-bones. The score details are as follows: 1: Emaciated—Obvious detection of ribs and other bones; 2: Thin—Easy detection of ribs and other bones while applying pressure; 3: Ideal—Ribs and other bones barely detectable while applying pressure; 4: Fat—No detection of ribs and other bones; and 5: Overly fat—No detection of ribs and other bones. 

Similar to the weight estimation, volume and growth were traditionally measured using tapes. Later, CCD cameras were used by Schofield et al. [30] to monitor pig growth. That study utilized a prototype that included two CCD to assess growth in 15 pigs. Earlier studies [17,32,50,59,60] achieved reliable 3D validation based on depth cameras compared to the 2D/CCD images. A depth-sensor-based mass estimation for growing and finishing pigs through extracting body shape information achieved 99% accuracy in the study by Condotti et al., 2018 [54], whereas the pig body surface was modeled in 3D based on the backbone area measurement and girth by Yoshida and Kawasue. [61] to sort the pigs according to the volume of the eventual carcass in the pig barn. An automatic pig size measurement system designed on the principle of the point cloud system that extracts body width, hip width, and body height with a lower relative error was developed by Wang et al. in 2018 [59]. That study developed a portable system that included an Xtion sensor and a PC to capture the overall pig image. Generally, an Xtion sensor consists of an infrared laser projector combined with two optical sensors for RGB imaging and depth sensing [59]. In the same way, open-source 3D point cloud analysis software for pig body measurement was released by Guo et al. [62] and is compatible with the Xtion prototypes; using this software, one can acquire body length, hip width, hip height, and heart girth information from pigs. Such systems are reliable to use at the farm level since they have been tested and implemented as prototypes.

### 4.4. Individual Pig Identification and Tracking

The study of the movement, behavior, and activities of pigs leads to their improved livelihood. The first step in tracking behavior is to identify an individual pig from the herd [63]. The current review covers behavioral identification, which includes aggression, posture, and locomotion; thus, it is necessary to have an accurate method of identifying individual pigs. Initially, individual pigs have been identified by RFID ear tags; we mentioned that this particular technology has limitations in terms of range and is expensive too. Camera-based computer vision techniques have proven to be a more promising solution for identifying a pig from a group in several studies [21,64,65,66,67]. In addition, such technologies are cost-efficient, involve no contact, and can be operated remotely. First, 2D gray/color cameras were most commonly used to identify a pig from a group; later, 3D and depth cameras were utilized for this purpose. The identification process has been performed with object detection from an image, then identifying the individual pig from the detected objects using morphological characteristics. Various studies used specific techniques such as GMM-based background subtraction [20,68,69], denoising using low-pass filtering followed by Otsu’s threshold method, morphological operations, ellipse fitting [21,28,64], graphical module-based segmentation [70,71,72], and learning-based tracking [65] for identification. 

Tracking/detection of a pig from a group using cameras has practical limitations. On a farm, there are illumination variations (the lighting conditions are not constant on farms), it can be challenging to find differences from one pig to another (sometimes pigs look identical from the top view), and there are object damage and barriers (uncertainty in tracking objects due to external interruptions such as insects or flies covering the lens). Such limitations cause tracking failures, and the continuous monitoring process becomes unstable. Zhang et al. and Sa et al. [66,73] successfully conducted studies to overcome such limitations using 2D and depth cameras. In [66], a 2D camera was utilized, and the detection of individuals pigs was conducted using CNN methods (Faster R-CNN, R-FCN, and SSD models were compared), whereas the tracking of the pig was performed with the bounding box method. The CNN-based model detects the individual pig in a tag box with a precision of 94.72% and a recall of 94.74%. A study to detect pigs quickly in various illumination conditions was conducted using a real-sense camera (depth and RGB camera) [73]. In that study, depth images, infrared images, and depth and infrared images were used to tackle the illumination limitations; thus, the study used Otsu’s threshold method for image segmentation and YOLO9000 for faster detection. Their proposed method was trained with mixed illumination images during the training and successfully validated with 95% real-time accuracy; moreover, the detection time was 8.71 ms/image, which is considerably lower than that of other CNN techniques.

## 5. Behavioral and Activity Detection 

### 5.1. Posture Detection

The welfare of an animal is evident in its behavior, physiology, health, and performance. Among other factors, behavior reveals the animal’s welfare right away, with its postures being a simple way to identify welfare. Pigs are diurnal animals by nature, spending 93% of the night asleep. Experts say they lie down for an average of 86% of the day [74,75]. Therefore, the lying-down posture of a pig is a critical factor, while other postures may be a sign of discomfort. The general posture name and the descriptions of postures are given in Table 1. The postures of pigs are considerably affected by non-thermal-neutral conditions (an inconsistent environment); for instance, a ±4 °C fluctuation in room temperature is sufficient to force a pig to move from its current lying posture to another, and an ±8 °C change had a significant effect on lying posture (*p* < 0.01) [74,75].

Furthermore, pigs are disciplined animals, using separate places for defecating and general use; this requires adequate space according to their body size. If not, homeostasis is affected. Therefore, as well as the environmental conditions, sufficient space notably affects the posture [76]. Several factors such as biological disturbance (insects and parasites) [77], floor type [78], nutrition plan [76], and radiation [77] are other aspects that affect the posture. Therefore, an effective mechanism for posture supervision is essential.

**Table 1:** Description of posture/behavior of a pig in an indoor farm collected from previous studies [19,77,79,80].

S. No.	Posture Name	Description
1	Sternal lying	Lying on the chest and belly on the floor; no visible legs due to them being folded under the body; totally hidden udder
2	Lateral lying	Lying sideways (on either right or left side); two or more legs visible; udder clearly visible
3	Standing	No other parts of the pig’s body except the legs are in contact with the floor; the end of the body should be visible
4	Sitting	Part of the body is in contact with the floor, with the front two legs stretched out; the end of the body is not visible
5	Feeding	Pig with head in food box/feeder for more than 2 s
6	NNV	Pig enters the feeding area with two or more feet, then leaves the feeding area having not consumed any food
7	Drinking	Pig touches one or more drinker for more than 2 s

Preprocessing techniques are necessary for object detection; cleaning the images, unwanted object removal, and applying filters are some preprocessing techniques used in previous studies, along with 2D images. Two decades ago, some studies [21,76,77,81,82] were conducted to determine thermal comfort by analyzing the posture of pigs using an ANN algorithm. Later, in 2008, a brief explanation of building real-time posture detection models was given by Shao and Xin [77]. In that study, images were segmented as frames from raw video files. The images were subjected to motion detection to confirm the movement of pigs in the current frame to the next frame using x and y coordinates. Generally, the threshold method was used to convert grayscale to a binary image. In contrast, blobs were introduced to remove small objects (manure) from the image before the feature extraction. Occupation ratio, run-length frequency, compactness, and two kinds of movement invariant were the features considered to determine the overall blob size according to temperature changes. Later, Nasirahmadi et al. [64,78,80] adopted Otsu’s threshold method, also known as the adaptive threshold, to convert binary [0,1] images to grayscale images. Unlike in the previous study, they used ellipse fit to locate the pigs. As a result, the properties of ellipse fit, i.e., major axis length, minor axis length, orientation, and centroid, were calculated to track the pig from the last frame. Later [80], they improved the lying posture method and classified the lateral and sternal lying posture using the SVM algorithm, while convex hull and boundaries were introduced to locate the pigs. That research successfully distinguished lateral and sternal lying postures with an accuracy of 94.2% and a sensitivity of 94.4%. After CNN become popular, several studies utilized CNN models such as TSN [66], DLM [83], YOLO5 [84,85], Resnet [84], and SCN [86] to perform the classifications. CNN algorithms are powerful and capable of multi-behavior recognition, so that particular studies increase the number of posture categories. Those studies also classified multiple postures of pigs with satisfactory results from 2D images/videos. 

Subsequently, depth-based 3D cameras were used for this purpose; a deeper analysis was conducted using depth-based sensors. For instance, an ethogram is a term used to distinguish signs of behavioral changes. A depth sensor-based behavioral study [19] was conducted by Matthews et al. in 2017 to predict behavioral changes in pigs. First, novel techniques were introduced to induce behavioral adaptations (treatment); then, without any distraction, normal day behavior (control) data were collected; both types of data were categorized into four groups to analyze the behavioral changes; and finally, using standard depth training methods, the point cloud data were trained and validated. Furthermore, a depth image-based study, especially for lactating sow postures using the Faster R-CNN algorithm, was conducted previously [79]. Unlike in other studies, the frequency of posture changes and the duration of every change (using timestamps) were monitored in that study. Lactating sow postures must be tracked to reduce the mortality rate of piglets. In that study, five categories of posture were classified from depth sensors with an accuracy of 93.5%; in addition, four time intervals were created, 20:00–23:59, 00:00–4:59, 05:00–11:59, and 12:00–19:59, for identifying posture distribution. By constantly tracking the centroid of the sow, a distribution map was generated. Furthermore, using a similar mechanism, the frequency of changing posture was calculated. This kind of study produces state-of-the-art research that can be used for analyzing and identifying significant changes in position for behavioral studies. 

### 5.2. Locomotion and Lameness

Locomotion is generally defined as the walking movement of pigs, whereas failure of locomotion is called lameness [16,87]. Locomotion tracking is similar to posture detection, but the categories would be measured in motion. Abnormality in locomotion is considered lameness, whether induced by the housing system, floor type, hygiene factors, genetics, toe or dewclaw management, or nutritional scarcity [16]. According to meat experts, damage to legs/lameness leads to average economic losses of $1.5 million/year [87]. Detecting locomotion is a way to detect lameness and initiate immediate treatment to reduce its severity. Identifying locomotion behavior using 2D cameras [27], IR camera [88], GoPro [89,90], or a web camera [26] was suggested by previous studies.

An automatic monitoring system for identifying locomotion was proposed in earlier studies [27,91]. A new approach was introduced to monitor the pigs’ motions; object marking was conducted by an ellipse fitting method, whereas objects were extracted using Otsu’s threshold for further processing. Segmented objects were fitted using ellipse-fitting methods with the default parameters (minor axis, major axis, centroid, and orientation). Later, image locomotion was used to determine the movement of pigs in terms of pixels. The image locomotion was calculated by the size of the movement vector in pixels (linear motion) with an angular vector (angular motion) in respect to the major axis (the body length of the pig). That image locomotion acquires the object movement in terms of the pixel with a threshold of 0.4 (if a pig moves more than 40% of his body length, the algorithm considers that the pig is moving). Then, the model was validated, and its accuracy compared with the eYeNamic tool (a successful model at that time); that study detected the locomotion of pigs with 89.8% accuracy. In [89], the walking movement of pigs was monitored using optical flow (OF) estimation and correction; such a study offers novelty in that OF methods had never been adopted for livestock before. That study collected video footage of pigs during a transfer from a truck to the pig house. Interestingly, a GoPro camera was used to take pig videos during unloading from fattening houses to the slaughterhouse. Optical flow estimation, identification of pigs, optical flow filtering, and distortion correction techniques were used for motion estimation. The pig movement was calculated using motion estimation, feature extraction, and frame classification. Four types of walking (free, encouraged, encouraged and tripping, and encouraged and stepping) were considered during the testing. SVM classifiers completed the group classification. This research suggests another way of collecting data rather than collecting conventional top-view data. An earlier study [26] proposed web-camera-based locomotion detection; in that study, multivariate image analysis (MIA) by principal component analysis was used, but MIA techniques were generally used to analyze the multispectral images. That study also filled standard frameworks until object segmentation, and then a movement filter was introduced to divide the pigs into moving and nonmoving. MIA techniques were used to gather information on moving frequency instead of wavelength.

Likewise, a Microsoft Kinect sensor, used to assess walking patterns in pigs, was studied by Stavrakakis et al. [92]. That study tried to prove that Microsoft Kinect sensors are capable of tracking pigs without any marks. Even though researchers use behavioral identification to ensure the pig health, pattern recognition is needed to set the thresholds. For instance, a previous study was conducted to find pig group lying patterns wherein a mathematical calculation named the Delaunay triangulation (DT) method was implemented in the machine vision algorithm. The DT method is usually used to track the patterns in a group of moving objects. According to the temperature changes, the pigs follow different structures. They change their patterns to avoid touching each other during high temperatures and huddle together to prevent the loss of body temperature in a colder environment. Sometimes the locomotion of a pig may change according to its lameness or any inconveniences. Research on locomotion remains lacking. 

### 5.3. Aggressive Behaviors

Pigs are social animals by nature, but their behavior is based on hierarchy while living in a group house. Aggression within a group is a common problem where a dominant–subordinate relationship is active [93,94,95,96,97]. The aggression of pigs is based on a destructive mindset of superior pigs against others and lasts from a few seconds to a few minutes. It can contribute to skin trauma, infection, fatal injuries, and sometimes death [93,97]. Both aggressive and injured pigs cannot access food properly, which leads to a reduction in their growth rate. Severe injuries provoke carcass contamination, which is a threat for economic reasons. Commonly, aggression type is divided into two groups: medium aggression (head-to-head knocking, head-to-body knocking, parallel pressing, inverse parallel pressing, and fleeing), and high aggression (neck biting, body biting, and ear biting). Even though many other factors such as individual aggressiveness and temperament, body weight, sex, and housing space come into play, aggression typically happens during the mixing of animals with an unfamiliar group [98].

In the early days, aggressive behavior surveillance was performed only by the human eye, a practice that requires significant human and material resources. Later, studies developed computer vision-based aggression behavior identification with 2D cameras [93,95,97] and 3D depth cameras [13,95,96,97]. The 2D camera-based feature extraction method for classifying aggressive behavior was proposed by Viazzi et al. [93]. That study considered that the duration of aggression was correlated with the contact between pigs, such that if the contact time of the two pigs was more than 5 s, that incident was counted as aggression. Motion history images (MHI) were generated using two 2D cameras placed in the pen, and the movement intensity of the pig was separated from the raw image. Secondly, attributes of the movement were extracted from the separated zones of MHI, whereas linear discriminative analysis (LDA) was utilized to classify aggression using a linear combination of features. The study successfully classified 133 aggressive interaction images from the 150 with 89% accuracy, 88.7% sensitivity, and 89.3% specificity. Likewise, another study [94] classified aggression behavior using ANN based on the activity scores. That study categorized the aggression intensity into medium and high, whereas the activity index was calculated by finding the number of pixels of moving animals. Five features (average, maximum, minimum, sum, and variance) were calculated over 14 different time intervals to set the activity scores. A multilayered feed-forward neural network was used to classify the behavior from the extracted features. The results had 96.1% sensitivity, 94.2% specificity, and 99.8% accuracy during high aggression classification, and 86.8% sensitivity, 94.5% specificity, and 99.2% accuracy during medium aggression. Similarly, a LDA and an ANN-based model for recognizing aggressive behaviors among group-housed pigs were conducted by a previous study [96]. Interestingly, the acceleration speed of pigs was extracted from the pixel movements to the next frame. That study considered that, if the movement of the pig is forceful, the aggression impact is high.

Depth-sensor-based aggression behavior identification studies have been conducted in recent years [95,97]. A trial was conducted to develop automatic recognition of aggressive behavior utilizing a Kinect depth sensor and a CCD camera [97]. That study defined aggression as head knocking and chasing. By using a depth camera, five features (the minimum, maximum, average, standard deviation of velocity, and distance between the pigs) were obtained. To classify the aggression, two SVM-based models were employed: SVM1 was used to identify the aggression detection from the depth image, and SVM2 was used to detect the category of aggression from the CCD camera image. Another study [97] utilized the SVM algorithm for aggression identification through depth and infrared images.

### 5.4. Tail Biting 

Tail biting is an acute problem in pig farming; it causes severe damage to animal welfare and production quality [99,100,101]. It causes stress due to pain but may also generate infection in the organs through wounds. Moreover, such conditions lead to partial or total contamination of the carcass quality, a vital parameter for economic aspects. According to pig production experts, a farmer may lose $1.10 per pig if the carcass is contaminated; in addition, wounds can increase the production cost by $0.59 per pig. However, the chances of tail biting in group-housed pigs are around 70% if the tail is visible [100,101]. Tail docking has been considered the standard solution for the last five decades to avoid tail biting. However, even though tail docking decreases tail biting, it is not totally effective [100]. There has not been much research on avoiding or detecting tail-biting using cameras. D’Eath et al. [100] proposed an early warning system for tail biting using a 3D camera.

As mentioned earlier [100], further research is needed for detecting tail-biting behavior with cameras. That study collected the symptoms of tail biting from the literature, and revealed four significant symptoms that may predict early tail biting. Those are:

1. Tail pointed down
2. Hyperactivity
3. Uncertainty of object-directed behavior
4. Outrage prevents victims from reacting.

That study used a top-view 3D camera in an enclosure for 667 pigs. Additionally, the tail posture was confirmed by the human eye using a 2D camera to compare with the machine vision results. Innovent Technology, Ltd. developed customized machine vision algorithms to locate the pigs and detect their tail postures. The validation consisted of four categories with different scoring for tail damage (0–4), wound freshness (0–5), tail length (0–3), and swelling (0–1). That study successfully detected the posture of the tail using 3D cameras based on early warning symptoms, with nominal accuracy. However, research about the other signs or methods for identifying tail biting remains limited. 

### 5.5. Eating and Drinking Solutions

Food and water are the primary necessities for living organisms. Unlike outdoor livestock, indoor livestock animals are unconditionally dependent on their farm owners for food and water. Numerous studies have shown that the growth rate is significantly affected if both are not available in the right proportions. Without proper nutrition, the animals can become depressed and are vulnerable to outbursts such as fighting among themselves [12,102,103,104,105,106,107]. Water and food are interdependent and linearly related; food behavior, especially, depends on water intake [105]. The eating habits of pigs determine their growth rate and indicate numerous characteristics such as their disease affect, early detection of disease, and mood [106]. Just as with the identification of pigs, feeding and drinking visits were also identified by conventional methods, which were RFID tags [12,106]. RFID receivers/readers were placed close to a feeder or drinker to collect the visiting time and duration information, and the collected data were analyzed. Setting up individual feeders is not an economical method; therefore, a cost-effective and reliable camera system is required. 

Non-nutritive visits (NNV) cause disputes among group-housed pigs. Pigs frequently visit the feeding area without consuming any feed, which may tempt others to rush to the feeding place [12]. This phenomenon increases the chance of damage through dashing. Changing such behavior requires adjusting the feeding plan. Even though there is a lack of research on monitoring the patterns of feed intake and duration of feeding time, feeding recognition was identified in behavior classification studies of pigs using cameras [19,107]. A study [19] was conducted to track behavioral changes in pigs with a 3D camera that differentiated between feeding and NNV. Two 3D cameras (depth and infrared) were placed to capture the whole day’s video to understand the pigs’ behavioral changes in the barn. NNV was considered as being when the pig enters the feeding area with two or more feet and then leaves the feeding area having not consumed any food, whereas a pig with its head inside of the feeder was considered to be feeding. Every day, the video footage was divided into 5 min blocks, and the XYZ coordinates of all animals were located in every frame. From the centroid of every pig marked in each frame, pigs were tracked using image processing by joining the centroids. Likewise, a depth-image-based study of lactating sow behaviors was conducted by Lao et al. [107]; they considered a sow to be feeding if she was moving her head in the feeder with an up and down movement. That study not only identified feeding; it also identified lying, sitting, standing, kneeling, feeding, drinking, moving, and shifting, with exceptional accuracy. Unlike in the studies mentioned above, some studies were specially designed to investigate feeding behavior. Yang et al. [12] and Alameer et al. [104] used 2D cameras and deep-learning methods. A previous study established the feeding verification of an individual pig from a group using a 2D top-view camera [12]. The head position attached to the feeder is considered a feeding phase. That study obtained the frames from the video sequences, and then pigs were individually detected from the group using bounty boxes. Later the head positions were identified for the training. Faster R-CNN models were utilized to detect whether the pig head was inside the feeder or not. Actual feed intake and foraging were distinguished in another study [104]. In addition, more detailed information on the type of feeding behavior was distinguished in that study; the categories were one pig feed intake, two pig feed intake, one pig NNV, two pig NNV, one pig feed intake and one pig NNV, two pig feed intake and one pig NNV, and no pig feed intake. That study followed a similar framework to the above study with promising results. 

One of the natural characteristics of a pig is enjoyment of being near water; therefore, it visits the drinker often in the same way as NNV. Most commercial pig farms choose one or more nipple drinkers for the water supply in one pen rather than bowls. The problem begins if a pig is playing in a nipple drinker; the other pigs have to put up a fight or remain thirsty [13,105]. To provide an adequate water supply for every pig, the drinking duration and number of visits should be monitored. A previous study observed the drinking duration and number of visits to the nipple drinker using a camera, but it was watched by human resources rather than automatically [105]. However, some studies were conducted to detect the drinking behavior through electronic methods [13,19,108,109]. An automatic monitoring system to determine the water use of pigs using an image-processing technique was proposed in a previous study [108]. CCD cameras were utilized for the collection of pig videos to extract images for preprocessing. The centroids of all the pigs were marked on the segmented image by calculating the maxima and minima of the object. The objects were identified by the ellipse fitting method. The possible area of drinking was marked in terms of pixels. By using single-input single-output (SISO), the extracted features were correlated and validated. In another study [13], the same method was followed to identify the pigs, whereas geometric features and color moment features were used to determine the drinking visits. That study confirmed that the pig was drinking by setting the duration threshold to over 2 s. A drinking study was also included in feeding and other behavioral studies; recent studies have detected drinking behavior using CNN techniques such as Faster R-CNN [19,109] and YOLO [109] with 2D cameras. 

Table 2 summarizes the camera type and methods employed by the previous studies selected for the current literature review. 

## 6. Early Disease Detection at a Farm Level

On a farm level, disease detection is an important measure to reduce and prevent diseases or pests in animals. Early disease detection is a practice to reduce the mortality rate through illness or infection. The following incident points out that the disease monitoring process cannot be ignored, especially in the fight against epidemics: in 2018, 200 million pigs were culled due to the swine flu outbreak in China [117,118] and 8 million pigs in Korea [102], which did catastrophic damage to the livestock industry. Disease detection is essential to ensure the health of pigs and prevent epidemics from transferring from pigs to humans. Testing the genotype materials frequently is impractical; correlating physical indicators is a solution for continual monitoring. In addition, noninvasive data collection or monitoring is the safest method for humans since the interaction between humans and animals can cause infection to pass from animals to humans or humans to animals. Generally, any infection causes weakness [114], fever [9,116], and progressive deceleration of diurnal activities [67]. Monitoring activities such as drinking, eating, lying, and walking is significant, and changes in activities are a way to track animal health. We have already discussed the identification and tracking of the behaviors of pigs; another significant indicator is the temperature of the pig’s body surface or parts. Since most infections cause a fever, body temperature fluctuation is an easy way to identify bovine viral diarrhea (BVD), *Salmonella typhimurium* infection, *Escherichia coli* infection, etc. [102]. The body temperature of pigs is variable in different body parts; each part, e.g., the eyes, nose, and legs, has a different normal temperature level [119,120]. Finding the temperature using infrared thermographic images is a successful method that has recently become standard. Moreover, giving activity scores over a 24 h period also points out infected pigs, and identifying mounting behaviors is helpful to avoid lameness [68].

Sweat glands are the temperature regulators for human beings, whereas pigs have no sweat glands to regulate the body temperature. Since disease causes an increase in the body temperature of pigs, infrared thermographic imagining (IRI) is an effective method to be aware of the temperature. A previous study [118] was conducted to assess the various body parts’ temperature using IRI. The following body parts were considered for temperature detection: the back of the ear, the lumbar area of the back, the sacral area of the back, the vulva, the lateral thigh, the mammary gland, the eye area, and the forehead. That study compared the accuracy between IRI and a regular infrared thermometer. However, that study required human resources; a later study sought to enable early disease detection by IRI [98,99,100,101]. Subsequently, utilizing IRI for early disease detection in pigs suffering from *Salmonella typhimurium* and *Escherichia coli* infection was proposed by a previous author [102]. A pig’s average daily gain was correlated with its temperature before infection and after infection by using thermal images from the literature. That study successfully distinguished the infections in terms of significant differences in the animal’s body temperature before and after. Moreover, 2D cameras are often employed for early disease detection; a lethargy detection study using computer vision techniques was also proposed in a previous study [113]. As we mentioned, disease decreases the regular activity of pigs; accordingly, this study considers lethargy as a way to identify African swine fever (ASF).

A couple of ASF-affected isolated pigs’ activities were monitored by cameras while another healthy pig’s activity was recorded for comparison. They used the CNN-based Alexnet for object identification and Kalman filter to track the behavioral metrics. They trained both videos and extracted the motion between the affected and healthy pigs for the whole day. Similarly, motion-based video monitoring for ASF detection was conducted in a previous study [112]. A typical 2D camera was used to record the videos; the experiment had four stages: ASF free (days 1–11), infection time (days 12–15), qPCR detection (days 16–18), and clinical detection (days 19–23). All the stages were completed. The motions were calculated by the optical flow method, and activities were classified as before and after infection. Likewise, an activity monitoring system for early disease detection was developed by Chung et al. [68], whereby they tracked the activity rhythm over a whole day. Concurrently, mounting behavior was key to predicting the lameness; for that, a previous study established a classification method for mounting behavior by using 2D cameras along with Mask R-CNN [114]. However, early disease detection remains a developing area in precision pig farming since disease is mostly caused by environmental factors. 

## 7. Discussion of the Real-Time Challenges and Limitations of Camera Sensors

**Figure 1:**
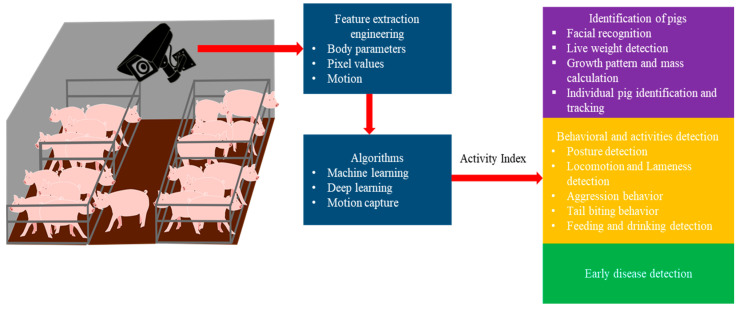
Camera sensors in pig barn for identification, activity detection, and early disease detection; a schematic of the current review.

**Figure 2:**
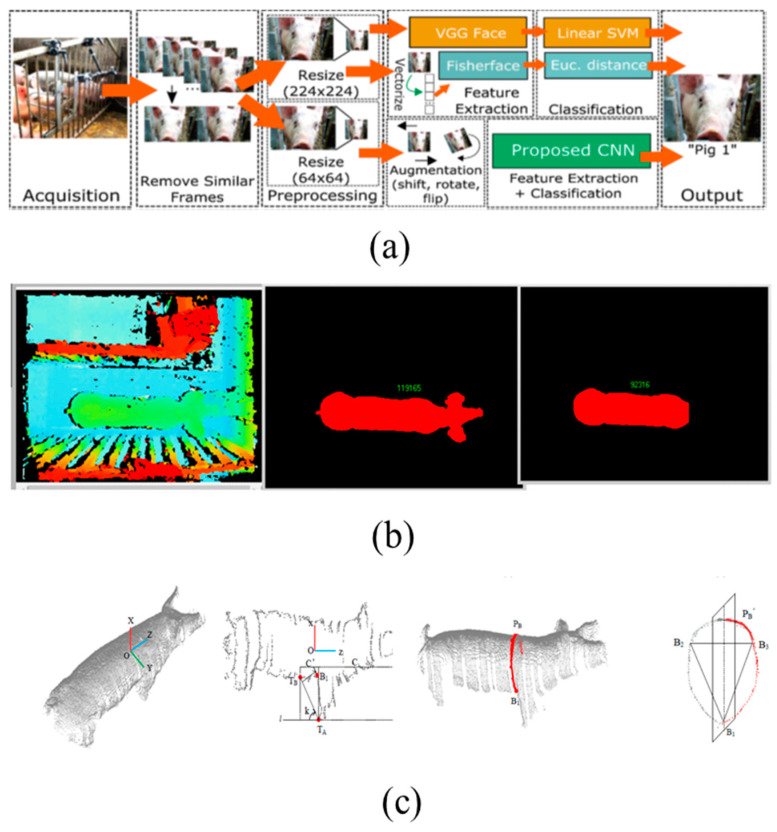
(**a**) Facial identification system proposed by Hansen et al. in 2018 [43]; (**b**) live pig weight estimation using a binocular stereo system based on LabVIEW by Shi et al. [53]; (**c**) heart girth measurement and detection of a pig using point cloud data, proposed by Zhang et al. in 2020 [32].

**Figure 3:**
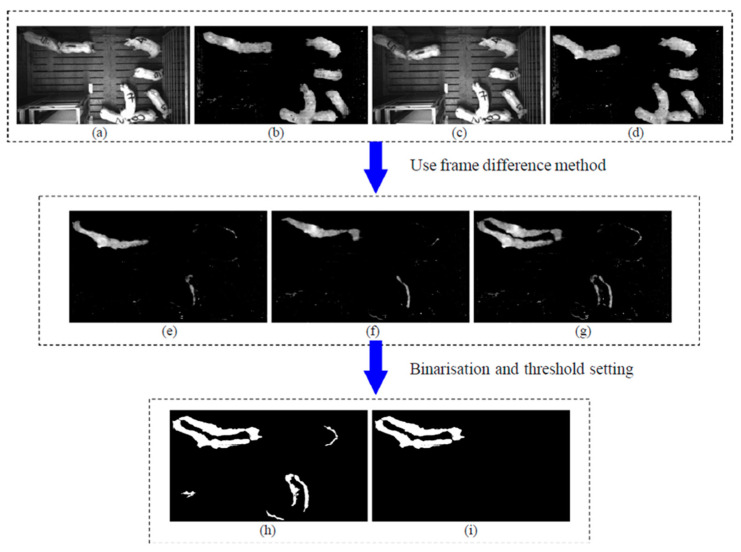
The extraction process of aggression-associated moving pixels: (**a**) former infrared frame, (**b**) former depth frame, (**c**) latter infrared frame, (**d**) latter depth frame, (**e**) latter minus former depth frame (I1), (**f**) former minus latter depth frame (I2), (**g**) extraction result for moving pixels (I1 + I2), (**h**) binarization result, and (**i**) extraction result for aggression-associated moving pixels after applying the threshold setting created by Chen et al. [95].

**Figure 4:**
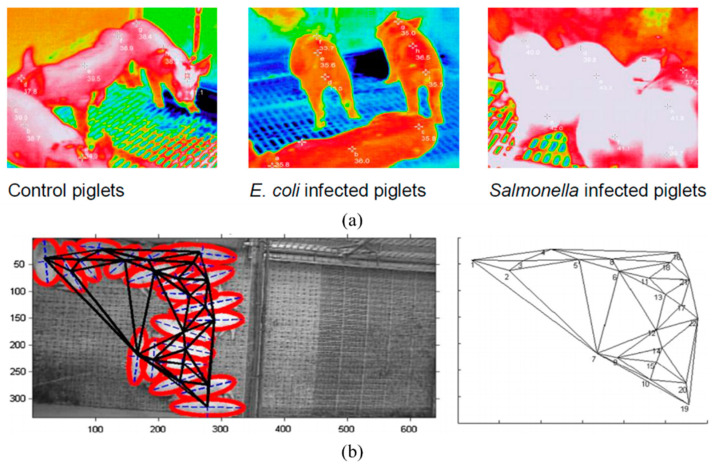
(**a**) Thermal images of control, *Salmonella enterica* serovar Typhimurium-infected, and *Escherichia coli*-infected pigs taken by Islam [102]; (**b**) Delaunay triangulation method to identify pig lying patterns by Nasirahmadi et al. [78].

Figure 1 explains the objective of the current study, whereas the methods/results of previous studies we used to improve our understanding are shown in Figure 2, Figure 3 and Figure 4. 

Even though technological advancement is rapid, it is not surprising that there are limitations on any technology. Similar to other PLF technology, camera sensors have some limitations that affect their implementation in commercial farms. Such limitations need to be tackled by future researchers to improve the efficiency of PLF. Most of the research selected for this review was conducted at a university, model pig barn, experimental sites, laboratory, and research institutes. The ground truth has enormous differences in those studies since the environment and ecology differ from one place to another. For instance, changes in the 8 °C indoor temperature significantly affect the huddling patterns, as mentioned above. Likewise, indoor room temperature plays a vital role in regulating pig body temperature and causes fluctuations in lying, eating, and drinking behaviors. Therefore, the difference between the model and the ground level is significant; there could be pseudo accuracy if a farmer adopts the same system for the experimental site and the actual pig barn. Indeed, the models have different accuracies according to the region, country, farm size, and breed; thus, the development of a global model is still lacking. In addition, every model should be validated by regional-level genetics since every species and its performance depends on the genetics, environment, regional climatic conditions, and food. A wild boar’s growth and characteristics are not similar to those of an American Yorkshire or Duroc breed pig; if a farmer uses the same algorithm, all the pigs would have false validation results. Even if researchers develop a global or regional model, they require data to train the algorithm; therefore, a farm needs to collect image data to update the model. In such cases, camera applications require a powerful computer, wireless communication, and the Internet; villages in developing countries still struggle for telecommunications access due to the business policies of telecom industries.

In most cases, a video camera should monitor the pigs constantly to flag up abnormal behavior. In this scenario, the monitoring system should generate many data points, but the amount of data is expensive and unprecedented. For instance, assume that a 2D camera is capturing video of a pigpen; if a 5 min video has a file size of 500 megabytes (for high-resolution cameras, it is even higher), this produces 141 gigabytes of visual data in a day, and approximately 4 terabytes per month. This greatly increases the operational costs of a farm. Bear in mind that IRI or other depth images require even more storage space than 2D images. It seems that optimal data-driven decisions depend on the advanced development of computer science.

The reliability of the validation results remains good if the results are compared with other useful analytical tools. In our literature review, the best studies utilized Masked R-CNN, Faster R-CNN models, YOLO, etc., for validation. However, every day, new algorithms with different optimizing techniques are being developed since most of the CNN models are available as open-source. Therefore, comparative studies should be conducted to optimize the algorithm and utilize the camera data more efficiently. For example, YOLOV5 is a recent machine-learning technique that is lightweight but powerful for identification; we realize how powerful and fast YOLOV5 is if we compare it with the other models. Thus, the performance of a particular algorithm is more reliable than that of a popular/previous model.

Even if an algorithm is feasible and reliable, it can only solve one of the problems that real farmers face if a 2D camera with a YOLOV5 algorithm can track the behavior of a pig but not the live weight or growth rate. So far, no studies have tried to design an algorithm to solve multiple problems. It seems that an integrated algorithm/system that can solve multiple issues is still lacking; thus, some studies should attempt to solve two or more problems with a simple framework. Subsequently, even though 2D cameras promise comparable and valid measurements compared to depth sensor-based prototypes such as Intel RealSense and Microsoft Kinect, 2D cameras cannot collect the maximum information from pigs. For example, a 2D top-view camera is not able to measure the height of pigs, whereas a side-view camera can only measure the breadth of a pig. Although integrating two or more 2D camera images to make a 3D point with computer vision can be a solution, the operational costs are higher than for depth sensors. Meanwhile, methods for using depth images to measure the body parameters of livestock are easy and inexpensive, but depth maps are sensitive to the illumination, background, and noise in ambient light; therefore, the calibration of such sensors becomes mandatory, and a high quality of images is required to produce desirable outcomes. Indeed, camera sensors are a simple, reliable, and inexpensive solution to improve quality and production in the pig industry. However, the limitations mentioned above should be considered in future studies to make the cameras an ultimate PLF implementation tool.

## 8. Conclusions

The current review summarizes studies utilizing 2D/3D/Infrared cameras to acquire information on pigs. In the future, farmers will depend on AI algorithms for automatic monitoring, control, and overall management. In developed countries, government spending is significantly investing in R&D for agricultural production. However, developing countries have no chance to invest more in agricultural technology. Technological development is the best solution to the food insecurity challenge in developing countries. To achieve this technical trade-off and improve food security, the knowledge gap between model implementation and real-time implementation should be diminished by researchers and practitioners. Moreover, we have discussed the practical limitations of camera sensors that explain why they are not considered the ultimate solution for medium- and small-scale livestock keepers. These limitations could be tackled through recent technological advancements such as drones, robots, etc. Solving multiple issues in one system boosts the adaption rate of PLF among farmers. In addition, fewer sensors, an affordable price, reliability, and easy handling are the factors that improve the production and quality. More studies need to come out to fill the knowledge gap in precision pig farming. We hope the information given in this review will be helpful for future researchers in building robust and balanced systems for pig farm management decisions. 

## Figures and Tables

**Table 2:** Summary of 2D and 3D camera algorithms used for precision pig farming from the reviewed studies.

Category	Parameters	Used Cameras	Used Tools and Techniques	Reference(s)
Identification	Face recognition	2D	Eigen space method	[44]
			CNN (SVM)	[43]
		Smartphone	Deep CNN	[40]
	Live weight	2D, CCD	Relating pixel values to morphological parameter dimensions	[6,29,30,50]
			ANN	[31]
			Transfer function model (by using body area)	[18]
			Vector-quantified temporal associative memory (VQTAM)	[45]
		Depth images	Relating pixel values to morphological parameter dimensions	[24]
			Machine vision module of the LabVIEW system	[53]
			Faster R-CNN	[51]
		2D (reconstructed as 3D)	Structure-from-Motion	[46]
	Growth and mass	CCD	Relating pixel values to morphological parameter dimensions	[29,60]
		3D, Xtion, Depth images	Extract pig body surface dimension parameters	[17]
			Relating pixel values to morphological parameter dimensions	[54]
			Point cloud method	[32,59,62]
			Point cloud method	[61]
	Individual identification	2D	Machine learning	[63]
			Ellipse fitting technique	[21,64]
			Gaussian mixture model	[20]
			R-CNN	[67]
			CNN (tag-box method)	[66]
			Adaptive partitioning and multilevel thresholding segmentation	[69]
			Machine vision (YOLOv3 and SORT)	[71]
			Blob detection	[28,72]
		Depth images	Image segmentation (Otsu’s thresholding)	[73]
Behavior and activities	Posture detection	2D	ANN (perceptron)	[76]
			SVM	[80]
			Faster R-CNN	[67,83]
			Ellipse fitting method	[20,80,110]
			Motion detection	[77]
			YOLOv5	[84]
			Two-stream convolutional networks	[85]
			Spatiotemporal convolutional network	[86]
		3D, Depth images	Otsu’s algorithm	[68]
			Faster R-CNN (bounding box)	[79]
			point cloud method	[19]
	Locomotion and Lameness	2D	CNN	[91]
			Ellipse fitting method, image locomotion	[27]
			Delaunay triangulation method	[64]
			Multivariate image analysis	[26]
		GoPro	Optical flow filtering	[89]
			Optical flow and angular histogram	[90]
		3D and Depth images	Motion capture	[92]
	Aggression	2D	Activity index	[94]
			Motion detection, Linear discriminant analysis	[93]
			Sequence extraction and motion analysis	[13]
		depth sensor	SVM	[97]
			Sequence extraction and motion analysis	[95]
	Tail biting	3D	Tail injury scoring by tail posture-detecting algorithm	[100]
	Feeding and drinking	2D	Transfer function modeling	[108]
			Object extraction	[13]
			Faster R-CNN	[12]
			CNN (Googlenet)	[104]
			CNN (YOLO)	[109]
		Depth image	Movement detection using centroids	[107]
			3D point clouds	[19]
Early disease detection	Abnormal behavior	2D	Activity monitoring for 24 h	[68]
	African swine Fever		Motion computation	[111,112,113]
	Mounting behavior		Mask R-CNN	[114]
	Infections from *Salmonella typhimurium* and *Escherichia coli*	Infrared thermography	Correlation between body temperature and average daily gain	[102]
	lameness		Temperature changes in leg area	[115]
	Body temperature changes		Gray–temperature conversion model (G–T model)	[116]
